# HIV-1 Tat Promotes Kaposi’s Sarcoma-Associated Herpesvirus (KSHV) vIL-6-Induced Angiogenesis and Tumorigenesis by Regulating PI3K/PTEN/AKT/GSK-3β Signaling Pathway

**DOI:** 10.1371/journal.pone.0053145

**Published:** 2013-01-02

**Authors:** Feng Zhou, Min Xue, Di Qin, Xiaofei Zhu, Cong Wang, Jianzhong Zhu, Tingting Hao, Lin Cheng, Xiuying Chen, Zhiqiang Bai, Ninghan Feng, Shou-Jiang Gao, Chun Lu

**Affiliations:** 1 State Key Laboratory of Reproductive Medicine, Nanjing Medical University, Nanjing, People’s Republic of China; 2 Key Laboratory of Pathogen Biology of Jiangsu Province, Nanjing Medical University, Nanjing, People’s Republic of China; 3 Department of Microbiology and Immunology, Nanjing Medical University, Nanjing, People’s Republic of China; 4 Department of Pathogenic Biology and Immunology, Xuzhou Medical College, Xuzhou, Jiangsu, People’s Republic of China; 5 Department of Physiology, Xuzhou Medical College, Xuzhou, Jiangsu, People’s Republic of China; 6 Pathology of Department, the First Affiliated Hospital of Nanjing Medical University, Nanjing, People’s Republic of China; 7 Cancer Virology Program, University of Pittsburgh Cancer Institute, Pittsburgh, Pennsylvania, United States of America; 8 Department of Urology, the First Affiliated Hospital of Nanjing Medical University, Nanjing, People’s Republic of China; 9 Department of Molecular Microbiology and Immunology, Keck School of Medicine, University of Southern California, Los Angeles, California, United States of America; Yale Medical School, United States of America

## Abstract

Kaposi’s sarcoma (KS)-associated herpesvirus (KSHV) is etiologically associated with KS, the most common AIDS-related malignancy. KS is characterized by vast angiogenesis and hyperproliferative spindle cells. We have previously reported that HIV-1 Tat can trigger KSHV reactivation and accelerate Kaposin A-induced tumorigenesis. Here, we explored Tat promotion of KSHV vIL-6-induced angiogenesis and tumorigenesis. Tat promotes vIL-6-induced cell proliferation, cellular transformation, vascular tube formation and VEGF production in culture. Tat enhances vIL-6-induced angiogenesis and tumorigenesis of fibroblasts and human endothelial cells in a chicken chorioallantoic membrane (CAM) model. In an allograft model, Tat promotes vIL-6-induced tumorigenesis and expression of CD31, CD34, SMA, VEGF, b-FGF, and cyclin D1. Mechanistic studies indicated Tat activates PI3K and AKT, and inactivates PTEN and GSK-3β in vIL-6 expressing cells. LY294002, a specific inhibitor of PI3K, effectively impaired Tat’s promotion of vIL-6-induced tumorigenesis. Together, these results provide the first evidence that Tat might contribute to KS pathogenesis by synergizing with vIL-6, and identify PI3K/AKT pathway as a potential therapeutic target in AIDS-related KS patients.

## Introduction

Kaposi’s sarcoma-associated herpesvirus (KSHV), also known as human herpesvirus 8 (HHV-8), is a γ_2_-herpesvirus. KSHV is linked to the development of Kaposi’s sarcoma (KS) [1], primary effusion lymphoma (PEL) and multicentric Castleman’s disease (MCD) [2]. KS is a vascular neoplasm of proliferative endothelial spindle cells. KS tumors contain abnormal and leaky vessels and extravasated red blood cells with haemosiderin deposits [3].

Although KSHV infection is necessary for the development of KS, it is not sufficient. The most important cofactor that contributes to KS development is HIV coinfection. The incidence of KS is 1 in 100,000 in the general population, but it is increased to 1 in 20 in HIV-infected individuals [4], and 1 in 3 in HIV-infected homosexual men before the introduction of HAART [5]. Higher KSHV infection rates and HIV-induced immune deficiency contribute to the higher KS incidence in the HIV population but they are likely not the only contributing factors. Previous studies have shown that KS are almost exclusively seen in HIV-1- but not HIV-2-infected individuals in Gambia, West Africa, despite similar KSHV infection rates and degree of immunodeficiency in both groups. Furthermore, KS often occurs early in AIDS, prior to the onset of severe immunosuppression. Additionally, AIDS-related KS (AIDS-KS) is more aggressive, disseminated, and resistant to treatment than iatrogenic KS. Therefore, additional factors could influence the development of AIDS-KS including secreted HIV-1 proteins, particularly Tat. Although HIV-1 does not infect KS tumor cells, studies have shown that Tat is readily detected in spindle cells of AIDS-KS lesions and promotes the growth of KS-derived endothelial cells (known as KS progenitor cells), thus might play a crucial role in the initiation and progression of KS in AIDS patients [6]–[8]. Our recent studies have revealed that Tat can not only activate lytic replication of KSHV by regulating the JAK/STAT signaling pathway [9], but also accelerate KSHV Kaposin A-induced cell proliferation and tumorigenesis [10].

KSHV encodes more than 90 genes and 25 mature miRNAs [11], many of which possess oncogenic properties [12]. Among them, vIL-6 encoded by ORF K2 is a homologue of cellular IL-6. Studies have demonstrated that vIL-6 can promote cellular proliferation, cell survival, and extrahepatic acute-phase response by stimulating several signaling pathways. vIL-6 engages the gp130 receptor but not the IL-6 receptor gp80 [13]. Furthermore, vIL-6 is expressed in 2∼5% PEL cells and 5∼25% B cells surrounding the follicular centers of MCD [2]. vIL-6 also contributes to KSHV immune evasion by inhibiting IFN-α-induced antiviral response [14]. In addition, vIL-6 can induce the secretion of cellular IL-6 and VEGF to promote cell proliferation of IL-6-depentent cell growth, and is required for hematopoiesis, angiogenensis and tumorigenesis [2], [15].

Although the mechanisms of KS pathogenesis by KSHV have not been fully clarified, several lines of evidence supported that the vGPCR plays a key role in KS initiation and progression. Recent studies have reported that PI3Kγ, a PI3K isoform exhibiting preferential expression in certain cell types such as endothelial cells (ECs), is strictly essential for vGPCR induction of AKT/mTOR signaling and sarcomagenensis [16]. Interestingly, by inducing activation of NF-AT and NF-κB, Tat accelerates vGPCR-induced tumorigenesis [17]. These observations have prompted to further investigate the interactions of Tat with other KSHV proteins.

In this study, we have revealed that HIV-1 Tat promotes vIL-6-induced angiogenesis and tumorigenesis in both chicken chorioallantoic membranes (CAM) model and an allograft model. We have shown that PI3K/PTEN/AKT/GSK-3β signaling is an essential pathway that mediates the process.

## Materials and Methods

### Ethics Statement

Animal care and handling conformed to the Guide for Care and Use of Laboratory Animals published by the US National Institutes of Health, and the study was approved by Nanjing Medical University’s ethical committee.

### Cells, Plasmids, Transfection and Reagents

NIH3T3, HEK293T and EA.hy926 cells were cultured as described previously [10], [18]. To express KSHV-vIL-6 in EA.hy926 and NIH3T3 cells, a 615-bp fragment of vIL-6 cDNA was amplified from BCBL-1 cells by PCR [15] and inserted into the pcDNA3.1 plasmid (Invitrogen Inc, Carlsbad, CA), named pK2-Flag. The overexpression plasmid PTEN cDNA construct (pPTEN) and the GSK-3β mutant plasmid GSK-3β-S9A (pS9A) were described previously [19]. The dominant-negative PI3K construct (PI3K-DN) and dominant-negative AKT construct (AKT-DN) were described elsewhere [19], [20]. The KSHV vIL-6 luciferase reporter construct (pvIL-6-Luc), which contains a 3.2-kb promoter region upstream of the methionine initiation codon of the vIL-6 gene, and RTA-expressing plasimid pcDNA-RTA were described previously [21], [22]. The plasmid pTZIII-CAT expressing the chloramphenicol acetyltransferase (CAT) enzyme under the control of the HIV-1 long terminal repeat (LTR) was described previously [9]. Transfections of EA.hy926 cells were performed with Effectence transfection reagent (Qiagen) and the other cells with Lipofectamine 2000 reagent (Invitrogen) according to the manufacturer’s instruction. The specific inhibitor of PI3K, LY294002, was purchased from Sigma (St. Louis, MO, USA). Recombinant full length HIV-1 Tat protein (101 amino acids) was purchased from Abcam (Cambridge, MA, USA).

### Generation of Stable Transfectants Expressing KSHV vIL-6

The study design is exhibited in Fig. S1. Briefly, to generate vIL-6-expressing KS-like tumor cell transfectant, the expression plasmid of pK2-Flag was transfected into NIH3T3 cells and the pcDNA3.1 vector was transfected for a negative control. The stable clones were obtained through selection by G418. Next, stable transfectant cells (5×10^6^ cells per 100 µl of PBS per animal) were injected subcutaneously (s.c.) into the left flank of male athymic BALB/c *nu/nu* mice. After four weeks, tumor tissue was detached, digested by trypsin and collagenase type 2 (Sigma) to obtain primary KS-like tumor cells. The primary cells were selected with G418 followed by end-point limiting dilution assay to acquire individual cell clones. The expression of vIL-6 mRNA and protein in primary tumor cell clones was detected by RT-PCR and Western blot, respectively. Finally, twelve stable transfectants were obtained; representative clones 4E3, 3D10, and the negative control T/V were used in this study.

Besides NIH3T3-derived KS-like tumor transfectant, endothelial cell (EC)-derived transfectant was also generated. As shown in Fig. S1, pK2-Flag was transfected into endothelial cells and the vector of pcDNA3.1 for a negative control. The individual stable clones were obtained through selection by G418 followed by end-point limiting dilution assay. Six stable transfectants were obtained by RT-PCR and Western blot; representative clones E6, F7, and the negative control E/V were used in this study.

### Lentiviral Tat Production and Transduction

The lentiviral plasmid system, the self-inactivating pHAGE-CMV-MCS-IzsGreen transferring plasmid, was used in this study. It was used to generate vesicular stomatitis virus (VSV)-pseudotyped lentiviral Tat, which would produce the full-length Tat101 of HIV-1[9]. A deletion mutant of Tat (△Tat), Tat△21–68, was also synthesized as a control. Tat△21–68 lacks both the cysteine-rich region of Tat, which is defective for transactivation of the HIV-1 long terminal repeat, and the induction region of neovascularization [23], [24]. The virus-packaging cells 293T (1×10^6^) were seeded in a 10-cm dish one day before transfection with pHAGE-Tat, packaging vector psPAX2 and envelope vector pMD2.G. Forty-eight hours after transfection, the virus containing supernatant was collected. The virus titres were determined on 293T to be about 2×10^7^ to 2×10^8^ transducing units (TU)/mL. The recombinant lentivirus full-length Tat, the deletion mutant of Tat and their vector control were designated as Tat, △Tat and Mock in this study, respectively.

### Cell Proliferation Assay

Cell proliferation was examined by MTT assays according to standard methods.

### CAT Assay

CAT assay was performed as described previously [9].

### Soft Agar Assay

Soft agar assay was performed as described previously [10]. Briefly, cell suspensions containing 0.3% agar were seeded in each well of 12-well plates containing an underlay of 0.6% agar in complete medium. Cultures were supplemented with complete medium per week and colonies were scored 14 to 21 days after seeding the cells.

### Microtubule Formation in Matrigel

Microtubule formation assay was performed on endothelial cells with the conditioned medium from 4E3 and T/V cells transduced by MOI 10 of lentivirus Tat or Mock for 48 hours as described previously [25].

### Chicken Chorioallantoic Membrane (CAM) Assay

White Leghorn fertilized chicken eggs were incubated at 37°C under constant humidity. To investigate the effect of Tat protein on KSHV vIL-6-induced angiogenesis, stable transfectant cells or cells isolated from induced tumor (5×10^5^ cells ) with or without transfection prior to transduction by MOI 10 of Tat or Mock were mixed at 1:1 ratio with Matrigel and implanted onto the chorioallantoic membranes (CAM) of chicken embryo at day 9. Tumor angiogenesis and tumor growth were analyzed 4 to 5 days after the implantation. The number and extent of branching of blood vessels were scored as relative angiogenesis index by two observers in a double-blind manner. The representative tumors were photographed.

### Tumorigenicity Assay in Nude Mice

The tumorigenic potential of stable transfectant with or without treatments was tested in 3- to 4-week-old male athymic BALB/c *nu/nu* mice (Shanghai Slac Laboratory Animal Center, Shanghai, China) housed under specific pathogen-free conditions. Cells (3×10^6^ cells per 100 µl of PBS per animal) were injected subcutaneously (s.c.) into the left flank. Five mice were used in each group, and experiments were performed twice. Tumor size was estimated by two-dimensional caliper measurement. For tumor allograft experiments, 4E3 cells transduced with Tat or Mock were injected s.c. into right flank of each mouse. When allografts reached volumes of 100 mm^3^ (about 10 days post-injection), the mice of Tat and Mock groups showing tumors were randomly split into a Mock+DMSO group, a Tat+DMSO group, a Mock+LY294002 group, and a Tat+LY294002 group. Accordingly, the groups were administered with either solvent control (DMSO) or LY294002 (25 mg/kg) by intraperitoneal injections (5 times, once for every two days).

### Western Blot and Antibodies

Western blot was performed as previously described [9]. Anti-VEGF (A20) rabbit polyclonal antibody (pAb), anti-basic FGF (b-FGF) rabbit pAb, anti-β-Actin mouse monoclonal antibody (mAb), anti-α-Tubulin mouse mAb, and horseradish peroxidase (HRP)-conjugated goat anti-mouse and rabbit IgG were purchased from Santa Cruz Biotechnologies (Santa Cruz, CA). Anti-Flag rabbit pAb, anti-cyclin D1 rabbit mAb, anti-phospho-PTEN (Ser380) rabbit pAb, anti-PTEN rabbit pAb, anti-phospho-PI3K [p85(Tyr458)/p55(Tyr199)] rabbit pAb, anti-PI3K rabbit pAb, anti-phospho-AKT (Ser473) mouse mAb, anti-AKT mouse mAb, anti-phospho-GSK-3β (Ser9) rabbit pAb, anti-GSK-3β rabbit pAb, anti-CD31 and CD34 mouse mAbs were obtained from Cell Signaling Technologies (Danvers, MA). Anti-smooth muscle actin (SMA) rabbit pAb were purchased from Abbiotec™ (San Diego, CA).

### Luciferase Reporter Assay

Luciferase reporter assay was performed as described previously [26].

### Immunohistochemistry

Informative sections of frozen or formalin-fixed, paraffin-embedded tumor from CAM or nude mice were immunostained as previously described [10]. Formalin-fixed paraffin-embedded tissue samples from one patient diagnosed with KS at the First Affiliated Hospital of Nanjing Medical University were obtained from the pathology archives for immunohistochemical studies.

### Statistical Analysis

All experiments were performed at least in triplicate if not mentioned. Numerical data were expressed as mean ± SD. Two group comparisons were analyzed by two-sided Student’s *t*-test. *P* values were calculated, and *P*<0.05 was considered significant.

## Results

### Tat Promotes Cell Proliferation and Vascular Tube Formation of vIL-6-expressing Fibroblasts and Endothelial Cells

**Figure 1:**
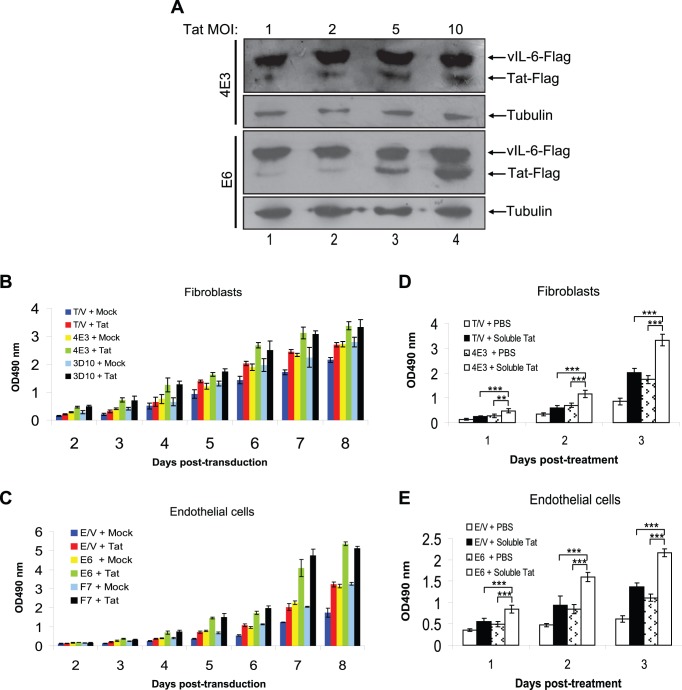
Tat promotes the proliferation of vIL-6 expressing cells. (**A**) Western blot analysis of Flag tagged vIL-6 and Tat expression in 4E3 and E6 cells transduced by different MOI of lentiviral Tat. (**B**) MTT assay for the proliferation of fibroblasts derived 4E3, 3D10 and control T/V cells and (**C**) endothelial cells derived E6, F7 and control E/V cells transduced by MOI 10 of Tat at days 2 to 8 post-transduction. The results represent the mean ± SD; shown is one representative experiment performed in triplicate. (**D**) MTT assay for the proliferation of fibroblasts derived 4E3, 3D10 and control T/V cells and (**E**) endothelial cells derived E6, F7 and control T/V cells stimulated with the soluble Tat (300 ng/mL) at days 1 to 3 post-treatment. **P<.01 and ***P<.001 for Student’s *t*-test versus corresponding controls, respectively.

KSHV vIL-6 induces angiogenesis and tumorigenesis when expressed in NIH3T3 cells [15]. We used 2 representative stable clones 4E3, 3D10 of vIL-6-transformed NIH3T3 tumor cells from the vIL-6-expressing tumors and 2 representative clones E6, F7 of vIL-6 expressing human endothelial cells in this study (Fig. S1). To investigate the interaction of vIL-6 with Tat, we transduced the cells with a lentivirus expressing Tat. Western blot showed that the expression level of Tat increased with MOI (Fig. 1A) without inducing apparent apoptosis and cell death (data not shown). As expected, vIL-6 was robustly expressed in both 4E3 and E6 clones but was not affected by Tat expression.

**Figure 2:**
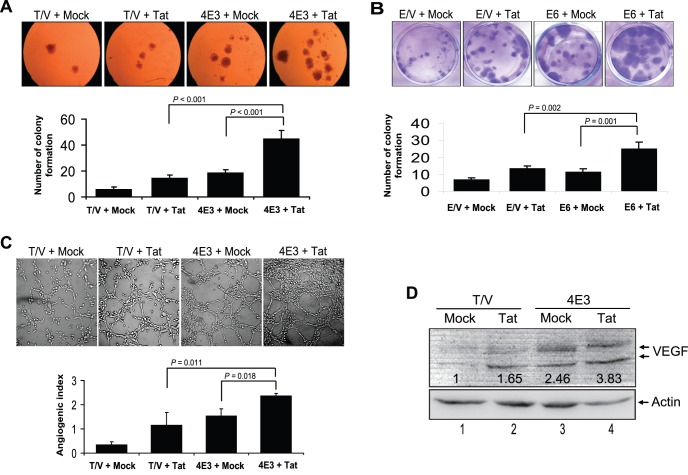
Tat promotes the transformation potential, microtube formation and VEGF production of vIL-6 expressing cells. (**A**) Soft agar analysis of the colony formation of 4E3 and control T/V cells and (**B**) plate colony formation assay for E6 and control E/V cells transduced by MOI 10 of Tat or Mock after two weeks. Photographs depict the colony formation (top; original magnification, ×100). The bars in the graph represent the mean ± SD; shown is one representative experiment of three performed (bottom). (**C**) Matrigel assay analysis of microtube formation. The photographs depict the microtubules after 6 hours of incubation (top; original magnification, ×100) and the quantified results represent the mean ± SD (bottom). (**D**) VEGF expression was analyzed by Western blot in 4E3 and T/V cells transduced by MOI 10 of Tat or Mock. The relative level of VEGF was determined by quantitative densitometry compared to actin. The relative value of VEGF in the T/V+Mock group was considered to be 1 for comparison.

We examined the effect of Tat on the proliferation of vIL-6-expressing cells using MTT assay. vIL-6-expressing cells 4E3 and 3D10 had higher proliferation rates compared to Mock cells (Fig. 1B). Expression of Tat further accelerated the proliferation of 4E3 and 3D10 cells. As expected, expression of Tat alone increased cell proliferation. Similarly, vIL-6 expressing endothelial cells E6 and F7 had higher proliferation rates compared to Mock cells (Fig. 1C). Expression of Tat further increased the proliferation of E6 and F7 cells. We next examined the effect of soluble Tat on the proliferation of vIL-6-expressing cells. Consistent with these results, soluble Tat accelerated the proliferation of vIL-6-expressing fibroblasts and endothelial cells (Fig. 1D and 1E), while it was functional in CAT assay (Fig. S2A and S2B). In soft agar assay, the number of colony formation of Tat-transduced 4E3 cells was significantly higher than that of Mock-transduced 4E3 cells (p<0.001) and Tat-transduced T/V cells (p<0.001), respectively (Fig. 2A). The similar results were also observed in vIL-6 and Tat co-expressing endothelial cells in plate colony assay (Fig. 2B), indicating that Tat enhanced vIL-6 cellular transformation potential. To examine the effect of Tat on vIL-6-induced angiogenesis, we performed tube formation assay. As shown in Fig. 2C, the tube formation of Tat-transduced 4E3 cells was significantly increased compared with those of both Mock-transduced 4E3 cells (p = 0.018) and Tat-transduced T/V control cells (p = 0.011). Examination of VEGF expression showed that the level of VEGF was correlated with the tube formation ability of the cells (Fig. 2D). Taken together, these data indicate that Tat promotes cell proliferation, cellular transformation and vascular tube formation of vIL-6-expressing cells.

### Tat Enhances vIL-6-induced Angiogenesis and Tumorigenesis

**Figure 3:**
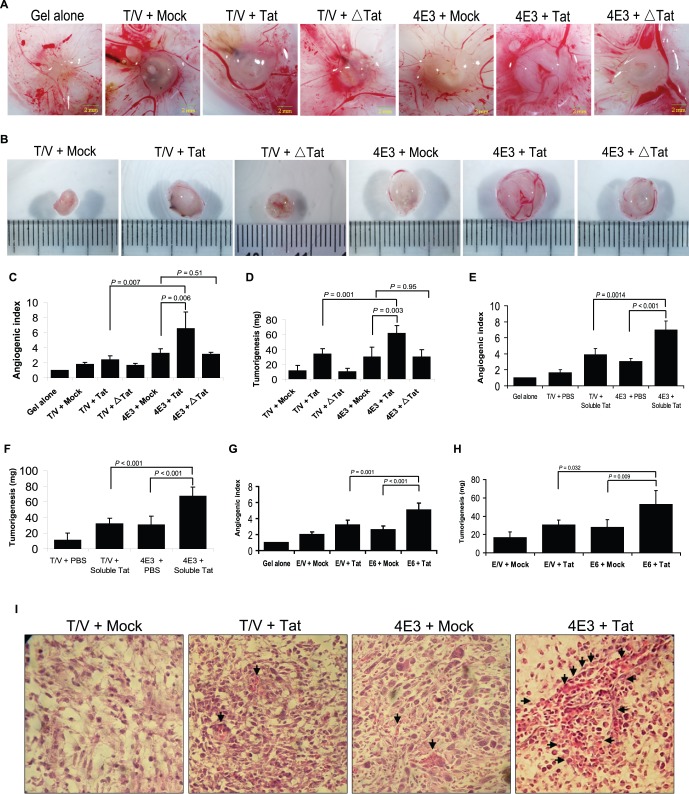
Tat enhances vIL-6-induced angiogenesis and tumorigenesis in the CAM model. (**A**) Tat enhances angiogenesis of 4E3 cells. 4E3 cells were transduced by lentiviral Tat, △Tat or Mock for 24 hours. Collected cells were mixed with matrigel and subsequently implanted onto the CAM (the detailed procedure seen in Methods). The representative angiogenic pictures and the quantification of blood vessels (**C**) on the CAM were shown. The number of blood vessels was normalized to that of Matrigel alone, and the results were expressed as the mean ± SD (n = 5 to 7). Two independent experiments were performed with similar results. (**B**) Tat enhances tumorigenesis of 4E3 cells. The representative tumor pictures and the quantification of tumor weight (**D**) on the CAM were shown. The tumor weight was normalized to that of Matrigel alone and the data represents the mean ± SD (n = 5 to 7). (**E**) Soluble Tat enhances angiogenesis and tumorigenesis (**F**) of 4E3 cells. The number of blood vessels was normalized to that of Matrigel alone, and the results were expressed as the mean ± SD (n = 6). (**G**) Tat promotes angiogenesis and tumorigenesis (**H**) of E6 cells. E6 cells were transduced by lentiviral Tat or Mock for 24 hours and implanted onto CAM. The tumor weight and number of blood vessels were normalized to those of Matrigel alone, and the data was the mean ± SD (n = 5 to 7). (**I**) H&E staining analysis of histological features in tumor tissues from Tat-transduced 4E3 cells on the CAM. The representative pictures were shown (original magnification, ×400). Arrows point to hemorrhagic foci.

We examined the effect of Tat on vIL-6-induced angiogenesis and tumorigenesis in the CAM model. 4E3 and T/V control cells were transduced by Tat or Mock and implanted onto the CAM. The angiogenesis index of Tat-transduced 4E3 cells increased significantly while compared with those of both Mock-transduced 4E3 cells (p = 0.006) and Tat-transduced T/V cells (p = 0.007) (Fig. 3A and 3C). Similarly, tumorigenesis ability of 4E3 cells was augmented by Tat compared with 4E3 cells transduced by Mock (p = 0.003) and T/V cells transduced by Tat (p = 0.001) (Fig. 3B and 3D). As expected, expression of Tat△21–68 alone did not increase the angiogenesis index or the growth rate of the tumor compared to Mock cells; and co-expression of Tat△21–68 and vIL-6 did not further increase angiogenesis and tumorigenesis. We next examined the effect of soluble Tat on vIL-6-induced angiogenesis and tumor formation. Consistent with above results, soluble Tat further increased angiogenesis and tumorigenesis of 4E3 cells in CAM model (Fig. 3E and 3F). Because KS tumors consist of predominantly endothelial cells [3], we employed stable vIL-6-expressing endothelial cells E6 for examination. Similar to fibroblasts, angiogenesis ability of Tat-transduced E6 cells increased significantly compared with those of both Mock-transduced E6 cells (p<0.001) and Tat-transduced E/V control cells (p = 0.001) (Fig. 3G). On the other hand, tumorigenesis ability of Tat-transduced E6 cells was augmented significantly compared with those of both Mock-transduced E6 cells (p = 0.009) and Tat-transduced E/V control cells (p = 0.032) (Fig. 3H).

H&E staining showed that tumors derived from the vIL-6 expressing cells were characterized by neovascularization, and various sizes and irregular shapes of hemorrhagic foci (Fig. 3I). These features were markedly enhanced in tumors derived from cells expressing both vIL-6 and Tat. Immunohistochemical staining showed increased expression levels of VEGF, b-FGF, and cyclin D1 in tumors from the Tat- and vIL-6-expressing cells, which were further enhanced in tumors from cells expressing both Tat and vIL-6 (Fig. S3A). These observations collectively demonstrated that Tat enhances vIL-6-induced angiogenesis and tumorigenesis of fibroblasts and endothelial cells.

### Tat Promotes vIL-6-induced Angiogenesis and Tumorigenesis by Regulating the PI3K/PTEN/AKT/GSK-3β Pathway

**Figure 4:**
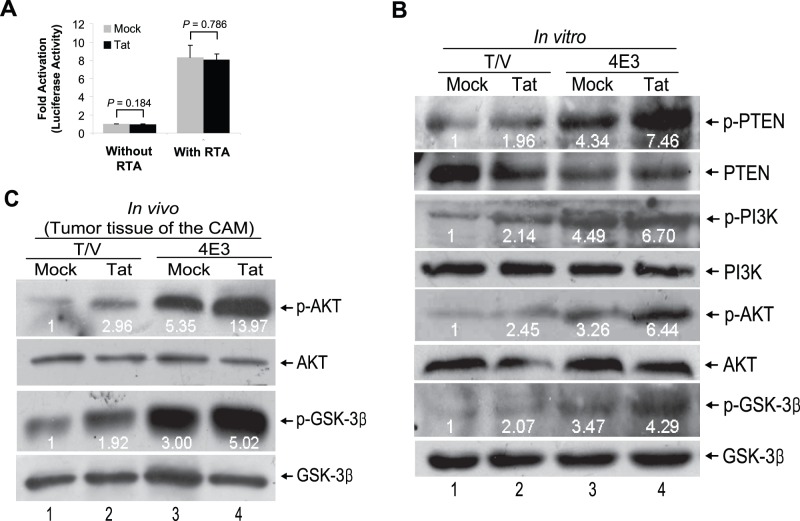
Tat augments vIL-6-induced phosphorylation of PTEN, PI3K, AKT, and GSK-3β both *in vitro* and *in vivo*. (**A**) Effects of Tat on vIL-6 promoter activities in 293T cells. Cells were transfected with RTA expression plasmid (**With RTA**) or its control vector (**Without RTA**) and subsequently cotransfected with the firefly luciferase reporter construct pvIL-6-Luc (0.4 mg) and a Renilla luciferase construct pRL-TK (0.02 mg) following infection with Tat or Mock. Luciferase activities were measured, normalized to inner control, and presented as fold increase (n-fold). All data points were the averages of three independent experiments performed in triplicate. (**B**) Phosphorylation levels of PTEN, PI3K, AKT, and GSK-3β were analyzed by Western blot in 4E3 and T/V cells transduced by MOI 10 of Tat or Mock. Both 4E3 and T/V cells were starved with serum-free medium for 24 hours and followed by Tat or Mock transduction for 24 hours. The relative level of phosphorylation protein was determined by quantitative densitometry after normalized to corresponding non-phosphorylation protein. The relative level of phosphorylation protein in T/V+Mock group was considered to be 1 for comparison. (**C**) Phosphorylation AKT and GSK-3β in tumor tissues from 4E3 and T/V cells on the CAM were analyzed by Western blot as above.

Because Tat is a *trans* activative transcription protein, we reasoned that it might influence vIL-6 transcription. Luciferase report assay was performed. We found that Tat failed to affect the promoter activity of vIL-6 either with or without expression of KSHV RTA (Fig. 4A), which was consistent with the above observation in vIL-6 protein expression (Fig. 1A). To dissect the mechanism of Tat promotion of vIL-6-induced angiogenesis and tumorigenesis, we further examined the PI3K/PTEN/AKT/GSK-3β signaling pathway. Consistent with the observed phenotypes, we found that expression of Tat or vIL-6 alone increased the phosphorylated forms of PTEN, PI3K, AKT, and GSK-3β in NIH3T3 cells (Fig. 4B). Expression of both Tat and vIL-6 further increased the levels of phosphorylated forms of these proteins. Of interest, the level of total PTEN was reduced in cells expressing Tat or vIL-6. Upregulation of phosphorylated AKT and GSK-3β was also observed in tumors (Fig. 4C).

**Figure 5:**
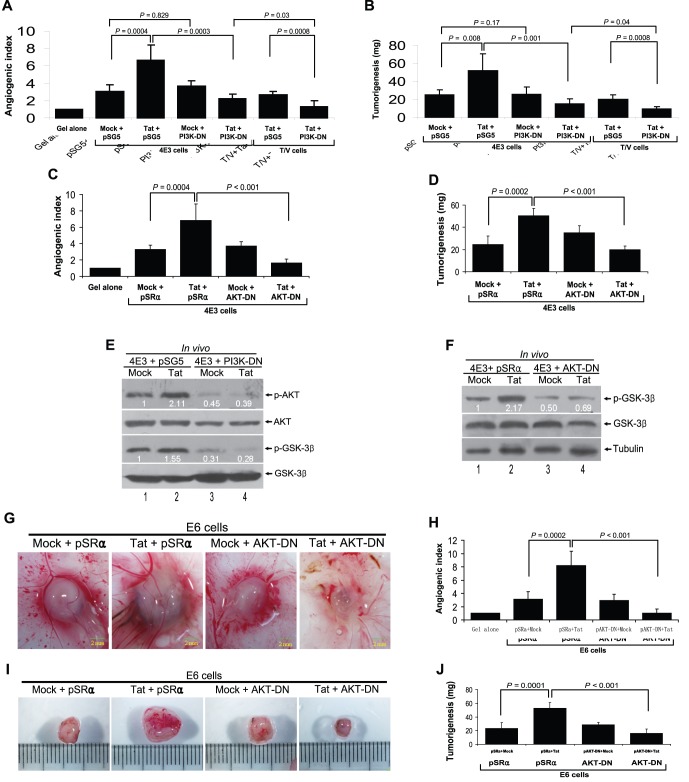
Tat promotes vIL-6-induced angiogenesis and tumorigenesis through activation of PI3K/AKT pathway in CAM model. (**A**) Inhibition of PI3K activity suppressed the enhanced effect of Tat on vIL-6-mediated angiogenesis. 4E3 or T/V cells were transfected by the dominant-negative PI3K construct (PI3K-DN) and its control plasmid pSG5 for 24 hours and followed by Tat or Mock transduction for another 24 hours. Collected cells were implanted onto the CAM. The quantification of blood vessels was expressed as the mean ± SD (n = 5 to 7). Two independent experiments were performed with similar results. (**B**) Inhibition of PI3K suppressed the enhanced effect of Tat on vIL-6-mediated tumorigenesis. 4E3 or T/V cells were treated as in (A). Quantification of tumor weight was expressed as the mean ± SD (n = 5 to 6). (**C**) Inhibition of AKT activity restrained the promoted effect of Tat on vIL-6-mediated angiogenesis. 4E3 cells were transfected by the AKT-DN and its control plasmid pSRα for 24 hours and followed by Tat or Mock transduction for another 24 hours. Collected cells were implanted onto the CAM. The quantification of blood vessels was expressed as the mean ± SD (n = 5 to 8). (**D**) Inhibition of AKT restrained the promoted effect of Tat on vIL-6-mediated tumorigenesis. 4E3 cells were treated as in (C). Quantification of tumor weight was expressed as the mean ± SD (n = 5 to 6). (**E**) Inhibition of PI3K abrogated the enhanced effect of Tat on phosphorylation of AKT and GSK-3β by vIL-6. 4E3 cells were treated as in (A). Collected cells were implanted onto the CAM. The phosphorylation levels of AKT and GSK-3β in tumor tissues of the CAM model were analyzed by Western blot. (**F**) Inhibition of AKT abolished the promoted effect of Tat on phosphorylation of GSK-3β by vIL-6. 4E3 cells were treated as in (C). Collected cells were implanted onto the CAM. The phosphorylation level of GSK-3β in tumor tissues of the CAM model was analyzed by Western blot. (**G**) Inhibition of AKT activity restrained the promoted effect of Tat on vIL-6-mediated angiogenesis of endothelial cells. E6 cells were transfected by the AKT-DN and its control plasmid pSRα for 24 hours and followed by Tat or Mock transduction for another 24 hours. Collected cells were implanted onto the CAM. The representative angiogenic pictures and the quantification of blood vessels (**H**) on the CAM were shown. (n = 6 to 8). (**I**) Inhibition of AKT restrained the promoted effect of Tat on vIL-6-mediated tumorigenesis of endothelial cells. E6 cells were treated as in (G). The representative tumor pictures and the quantification of tumor weight (**J**) on the CAM were shown. The tumor weight was normalized to that of Matrigel alone and the data represents the mean ± SD (n = 6).

We further determined whether Tat enhanced vIL-6-induced angiogenesis and tumorigenesis is regulated by the PI3K/PTEN/AKT/GSK-3β pathway. Expression of a dominant negative mutant of PI3K completely inhibited Tat-mediated enhancement of angiogenesis and tumorigenesis (Fig. 5A and 5B). Similar results were also observed with a dominant negative mutant of AKT (Fig. 5C and 5D). As expected, inhibition of PI3K resulted in the reduction of phosphorylated forms of AKT and GSK-3β, both of which are downstream of PI3K (Fig. 5E). Inhibition of AKT resulted in the reduction of phosphorylated forms of GSK-3β, which is downstream of AKT (Fig. 5F). Histologically, inhibition of AKT not only decreased the tumor features including hemorrhagic foci and neovascularization (Fig. S3B), but also reduced the levels of VEGF, b-FGF, and cyclin D1 (Fig. S3C).

We further confirmed the above observations in endothelial cells. Similar to the results in Fig. 5C and 5D, the quantification of both blood vessels and tumor weight showed that inhibition of AKT activity suppressed the promoting effect of Tat on vIL-6-induced angiogenesis and tumorigenesis of endothelial cells in the CAM model (Fig. 5G, 5H, 5I, 5J).

**Figure 6:**
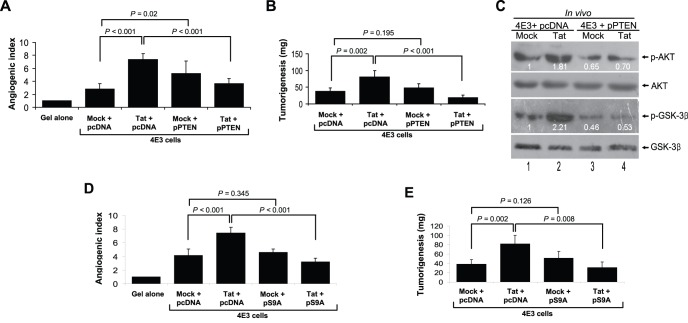
Tat enhances vIL-6-induced angiogenesis and tumorigenesis through inactivation of PTEN and GSK-3β signals in CAM model. (**A**) Overexpression of PTEN suppressed the enhanced effect of Tat on vIL-6-mediated angiogenesis. 4E3 cells were transfected by pPTEN and its control plasmid pcDNA for 24 hours and followed by Tat or Mock transduction for another 24 hours. Collected cells were implanted onto the CAM. The quantification of blood vessels was expressed as the mean ± SD (n = 5 to 8). Two independent experiments were performed with similar results. (**B**) Overexpression of PTEN suppressed the enhanced effect of Tat on vIL-6-mediated angiogenesis. 4E3 cells were treated as in (A). Quantification of tumor weight was expressed as the mean ± SD (n = 5). (**C**) Overexpression of PTEN restrained the enhanced effect of Tat on phosphorylation of AKT and GSK-3β by vIL-6. 4E3 cells were treated as in (A). Collected cells were implanted onto the CAM. The phosphorylation levels of AKT and GSK-3β in tumor tissues of the CAM model were analyzed by Western blot. (**D**) Overexpression of GSK-3β decreased the promoted effect of Tat on vIL-6-mediated angiogenesis. 4E3 cells were transfected by the pS9A and its control plasmid pcDNA for 24 hours and followed by Tat or Mock transduction for another 24 hours. Collected cells were implanted onto the CAM. The quantification of blood vessels was expressed as the mean ± SD (n = 5 to 8). (**E**) Overexpression of GSK-3β decreased the promoted effect of Tat on vIL-6-mediated tumorigenesis. 4E3 cells were treated as in (D). Quantification of tumor weight was expressed as the mean ± SD (n = 5).

Because the phosphorylated PTEN was elevated in Tat-transduced 4E3 cells (Fig. 4B), we expressed PTEN (pPTEN) in these cells and assessed the effect on angiogenesis and tumorigenesis. Expression of pPTEN not only significantly inhibited Tat-mediated enhancement of angiogenesis and tumorigenesis (Fig. 6A and 6B) but also decreased the enhanced effect of Tat on the phosphorylation of AKT and GSK-3β by vIL-6 (Fig. 6C). The above results showed that activation of PI3K and AKT resulted in the inhibition of GSK-3β (Fig. 4B and 4C) indicating that GSK-3β might mediate Tat-induced enhancement of angiogenesis and tumorigenesis. Indeed, expression of GSK3β-S9A (pS9A), a GSK3β mutant, inhibited Tat-mediated enhancement of both angiogenesis and tumorigenesis (Fig. 6D and 6E).

Together these data suggest that Tat augments vIL-6-induced angiogenesis and tumorigenesis by activating PI3K/AKT and inactivating PTEN and GSK-3β signals in both fibroblasts and endothelial cells-mediated CAM model.

### Activation of PI3K/AKT Pathway is Required for Tat Promotion of vIL-6-induced Tumorigenesis

**Figure 7:**
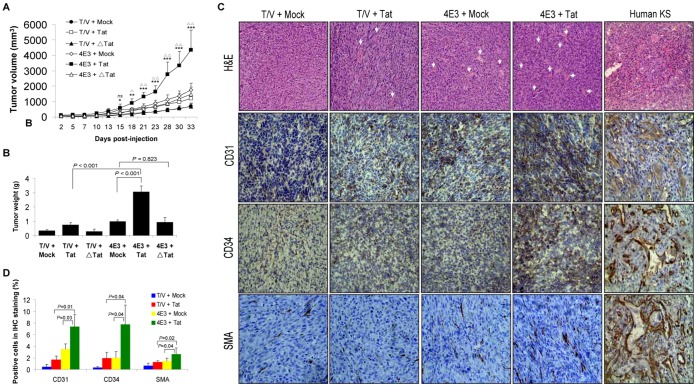
Tat promotes vIL-6-induced tumorigenesis in nude mice. (**A**) Plot of the volume of the tumors. 4E3 and T/V cells were transduced by Tat, △Tat or Mock and injected s.c. into the left flank of nude mice. The animals were monitored every day for the appearance of tumors until 33 days. Tumor size was estimated by two-dimensional caliper measurement. The results are expressed as the mean ± SD (n = 5). *P<.05, **P<.01 and ***P<.001 for Student’s *t*-test versus T/V+Tat groups, respectively. ^△^ *P*<.05 and ^△△^ *P*<.01 for Student’s *t*-test versus 4E3+ Mock groups, respectively. *ns*, not significant, *t*-test. (**B**) Column diagram of average tumor weight. Tumor-bearing mice were killed at day 33 after injections; and tumors were removed and weighed. Data reflect the mean ± SD. (**C**) H&E staining analysis of histological features and immunohistochemical staining analysis of the expression levels of CD31, CD34 and SMA in tumor tissues from Tat-transduced 4E3 cells in nude mice. Both human KS sample and nude tumor samples for SMA detection were formalin-fixed and paraffin-embedded sectioned. The rest were frozen sections. The representative pictures were shown (original magnification, ×200). Arrows point to hemorrhagic foci. (**D**) Quantification of results in (D).

**Figure 8:**
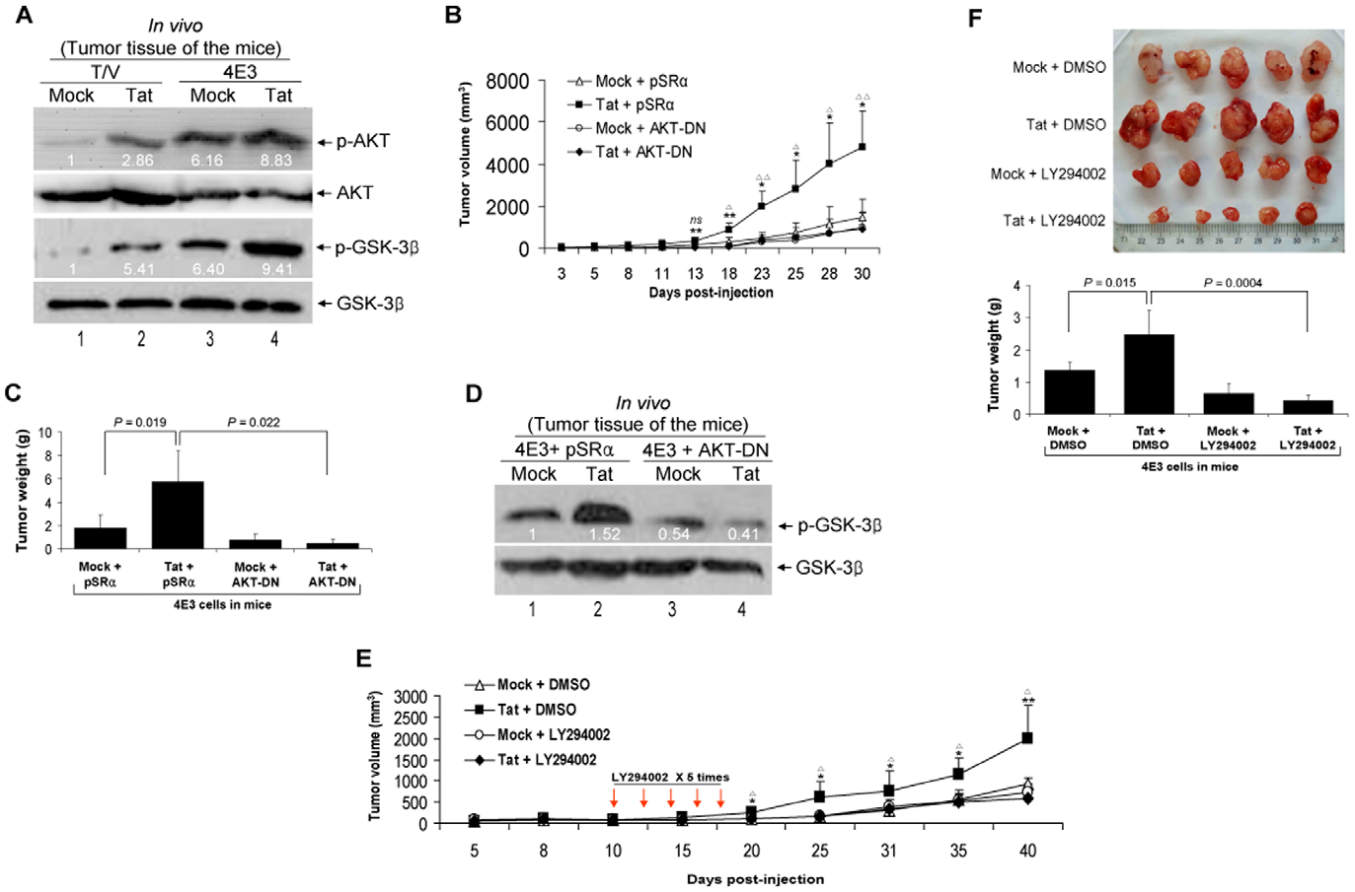
Activation of PI3K/AKT pathway is required for promotion of vIL-6-induced tumorigenesis by Tat in nude mice. (**A**) Tat augments vIL-6-induced phosphorylation of AKT and GSK-3β in nude mice. Phosphorylation AKT and GSK-3β in tumor tissues from 4E3 and T/V cells transduced by Tat and Mock in nude mice were analyzed by Western blot. (**B**) Plot of the volume of the tumors. 4E3 cells were transfected by the AKT-DN and its control plasmid pSRα for 24 hours and followed by Tat or Mock transduction for another 24 hours. Cells were injected s.c. into the left flank of the mice. The animals were monitored every day for the appearance of tumors until 30 days. Tumor size was estimated by two-dimensional caliper measurement. The results are expressed as the mean ± SD (n = 5). *P<.05 and **P<.01 for Student’s *t*-test versus Tat+AKT groups, respectively. ^△^ *P*<.05 and ^△△^ *P*<.01 for Student’s *t*-test versus Mock+pSRα groups, respectively. *ns*, not significant, *t*-test. (**C**) Column diagram of average tumor weight. Tumor-bearing mice were killed at day 30 after injections, and tumors were removed and weighed. Data reflect the mean ± SD. (**D**) Inhibition of AKT activity abolished the promoted effect of Tat on phosphorylation of GSK-3β by vIL-6. 4E3 cells were treated as in (B). The phosphorylation level of GSK-3β in tumor tissues of nude mice was analyzed by Western blot. (**E**) Plot of the volume of the tumors. 4E3 cells transduced with Tat or Mock were injected s.c. into mice for allograft formation. The mice received the treatments by intraperitoneal injection of LY294002 (the detailed procedure seen in Methods). The results are expressed as the mean ± SD (n = 5). *P<.05 and **P<.01 for Student’s *t*-test versus Tat+LY294002 groups, respectively. ^△^ *P*<.05 for Student’s *t*-test versus Mock+DMSO groups, respectively. (**F**) Column diagram of average tumor weight. Tumor-bearing mice were killed at day 40 after injections, and tumors were removed (Top) and weighed. Data reflect the mean ± SD (Bottom).

We further examined the effect of Tat on the growth of vIL-6-induced tumors in nude mice. Expression of vIL-6 or Tat alone moderately accelerated the growth of tumors induced by NIH3T3 cells. However, expression of both Tat and vIL-6 significantly increased the tumor growth rates (Fig. 7A). At 33 days post-inoculation, the average tumor weight was strikingly higher with the Tat-transduced 4E3 cell group compared to that of by Mock-transduced 4E3 cell group or T/V control cells transduced by Tat alone (Fig. 7B). As expected, expression of Tat△21–68 failed to accelerate the growth of tumors and increase the average tumor weight by vIL-6 (Fig. 7A and 7B). Histologically, the tumor was characterized by various sizes and irregular shapes of dense neovascularization and hemorrhagic necrotic foci (Fig. 7C). Large multinucleated cell infiltrations of lymphocytes were present in the tumors. These histological features were increased in the Tat-transduced 4E3 cell group compared to Mock-transduced 4E3 cell group or T/V control cells transduced by Tat alone (Fig. 7C). Immunohistochemical staining showed the expression of CD31, CD34, SMA, VEGF, b-FGF, and cyclin D1 in tumors which were substantially increased in Tat-transduced 4E3 cells (Fig. 7C and 7D; Fig. S4A and S4B). Western blot with extracts from the tumors showed increased levels of phosphorylated forms of AKT and GSK-3β in the Tat-transduced 4E3 cell group compared to those of 4E3 cells transduced by Mock and T/V control cells transduced by Tat (Fig. 8A), indicating the involvement of AKT signaling in Tat-mediated promotion of vIL-6-induced tumorigenesis. Expression of a dominant negative mutant of AKT (AKT-DN) but not its vector control (pSRα) inhibited the progression of the tumors of Tat-transduced 4E3 group (Fig. 8B). Examination of the tumor weight at day 30 post-inoculation showed that cells expressing AKT-DN had substantially smaller tumor sizes compared to vector control (Fig. 8C). H&E staining showed that the numbers of newly formed vessels and scattered lymphocytes were markedly reduced in Tat-transduced 4E3 cells expressing AKT-DN compared to the vector control (Fig. S5A). Immunohistochemical staining demonstrated that the expression levels of VEGF, b-FGF, cyclin D1, and SMA were reduced in tumors induced by Tat-transduced 4E3 cells expressing AKT-DN compared to vector control (Fig. S5B and S5C). As expected, AKT-DN also reduced the level of the phosphorylated form of GSK-3β compared to the vector control (Fig. 8D). These results indicated that activation of AKT mediates Tat promotion of vIL-6-induced tumorigenesis.

To determine the role of PI3K in Tat-mediated promotion of vIL-6-induced tumors, we treated the tumor-bearing mice with LY294002, a specific PI3K inhibitor for 5 times at the indicated times starting on day 10 post-inoculation. Similar to AKT-DN, LY294002 effectively inhibited the growth of the tumors induced by Tat-transduced 4E3 cells compared to tumor-bearing mice treated with DMSO (Fig. 8E and 8F). Taken together, these results indicated that PI3K is an effective target for the vIL-6 and Tat-induced tumors.

## Discussion

Previous studies have shown that vIL-6 can directly bind to the gp130 receptor on human, mouse and rat cells, thereby activating the JAK/STAT signaling pathway. Therefore, vIL-6 mimics biological properties of cellular IL-6, such as supporting cell growth of the IL-6-dependent cell lines B9 or INA-6 and inducing acute-phase gene expression in a hepatocellular carcinoma cell line [27]–[30]. However, in contrast to IL-6, vIL-6 does not require the IL-6R for receptor complex formation and signaling. Accordingly, vIL-6 can stimulate far more cell types than IL-6 can since its activity is not restricted to cells that express the IL-6R. Furthermore, the vIL-6-producing NIH3T3 cells give rise to tumors in nude mice more quickly than control cells do [15]. Here, we revealed that vIL-6 expressing NIH3T3 cells induced vast angiogenesis and tumorigenesis and vIL-6 also promoted angiogenesis and tumorigenesis induced by endothelial cells in the CAM model. Together, these results highlight the oncogenic properties of the vIL-6 protein and its likely important role in KS pathogenesis.

We and others have demonstrated that Tat possesses multiply biological activities including activation of KSHV replication [9], [31], enhancement of cell proliferation [32]–[35], induction of KS-like lesions [4], [36], and acceleration of tumorigenesis by vGPCR and Kaposin A [10], [17]. These results suggest that Tat plays a vital role in AIDS-KS pathogenesis, however, the underlying molecular mechanism remains unclear. Here, our study, for the first time, has directly tested the effect of Tat on angiogenesis induced by KSHV vIL-6. The synergistic effect that we have observed is very striking. As uptake of Tat by cells is very efficient [34], [37], Tat is mostly positive in spindle cells of AIDS-KS lesions [8], and HIV-1-infected patients often have high level of circulating Tat [38], we believe that our observations are highly relevant to the clinical setting.

Our results also showed that Tat did not directly increase the expression of vIL-6, suggesting that its primary impact on vIL-6-induced angiogenesis and tumorigenesis may be due to direct activation of cellular signal(s) and/or enhancement of vIL-6 signaling. The PI3K/AKT signaling axis plays an important role in cellular proliferation, cell survival, neovascularization, and tumor growth. Several components of this signaling axis were dysregulated in many cancers, including KS [39]–[42]. Activated AKT triggers downstream mTOR/p70S6K1 signaling resulting in the induction of pro-angiogenic factors such as VEGF and b-FGF, thereby inducing neovascularization to promote tumor growth [43]. On the other hand, activated AKT phosphorylates and inactivates GSK-3β to decrease phosphorylation of cyclin D1, and accordingly, triggering cell proliferation [44]. Notably, PI3K/AKT axis can be modulated by PTEN, a tumor suppressor which removes the 3′phosphate of PIP3 and attenuates signaling downstream of the activated PI3K. With regard to relationship between PI3K/AKT and vIL-6, we found that PI3K/AKT activated while PTEN/GSK-3β was inactivated in both vIL-6-producing 4E3 cells and 4E3-mediated tumor tissues from CAM and nude mice models. Inhibition of PI3K/AKT or overexpression of PTEN or GSK-3β failed to impair vIL-6 induction of angiogenesis and tumorigenesis in CAM and nude mice allograft models, indicating that vIL-6 might exert its biological function mainly via JAK/STAT signal rather than PI3K/AKT signal [27], [29], [30]. Therefore, we concluded that activation of PI3K/AKT pathway and inactivation of PTEN/GSK-3β signal were likely not the main cause of vIL-6-induced angiogenesis and tumorigenesis.

Although Tat could activate PI3K/AKT-dependent survival pathways in KS cells [45], in this study, we showed that in Tat-transduced vIL-6-expressing cells, inhibition of PI3K/AKT and overexpression of PTEN or GSK-3β signal efficiently suppressed the enhanced effect of Tat on vIL-6-induced angiogenesis and tumor development *in vivo*. Moreover, the PI3K inhibitor LY294002 greatly impaired the ability of Tat to promote vIL-6-induced tumorigenesis in allograft model. These data illustrated that Tat enhanced vIL-6-induced angiogenesis and tumorigenesis by regulating PI3K/PTEN/AKT/GSK-3β pathway. Therefore, PI3K/AKT may represent an attractive therapeutic target for patients with AIDS-related KS. Besides PTEN/AKT signaling axis, Tat can also activate other multiply cellular signaling pathway [7], [9], [10], [46]. Thus, additional mechanism by which Tat regulates vIL-6-induced angiogenesis and tumor growth remains possible.

## Supporting Information

Figure S1 **Schematic diagram for generation of stable transfectants expressing vIL-6, including NIH3T3-derived KS-like cell clones expressing vIL-6 and endothelial cell clones expressing vIL-6.**
(EPS)

Figure S2 **ELISA for CAT in 4E3, E6, and their corresponding control cells transfected with pTZIII-CAT and followed by lentiviral Tat transduction (A) or soluble Tat treatment (B) for 48 hours.** CAT protein expression was quantitated by ELISA. Results presented were from three independent experiments performed in triplicate.
(EPS)

Figure S3 **Tat augments vIL-6-induced tumorigenesis, leading to VEGF, b-FGF and Cyclin D1 expression in CAM model.** **(A)** Immunohistochemical staining analysis of the expression levels of VEGF, b-FGF, and cyclin D1 in tumor tissues from Tat-transduced 4E3 cells. The representative pictures were shown (original magnification, ×400). **(B)** H&E staining analysis of histological features in tumor tissues from AKT-DN-transfected 4E3 cells followed by Tat transduction in the CAM model. The representative pictures were shown (original magnification, ×400). Arrowheads point to hemorrhagic foci. **(C)** Immunohistochemical staining analysis of the expression levels of VEGF, b-FGF, and cyclin D1 in tumor tissues from AKT-DN-transfected 4E3 cells followed by Tat transduction in the CAM model. The representative pictures were shown (original magnification, ×400).
(TIF)

Figure S4 **Tat augments vIL-6-induced tumorigenesis, leading to VEGF, b-FGF and Cyclin D1 expression in nude mice. (A)** Immunohistochemical staining analysis of the expression level of VEGF, b-FGF, and cyclin D1 in nude mice tumor tissues from Tat-transduced 4E3 cells. The representative pictures were shown (original magnification, ×400). **(B)** Quantification of results in (A).
(EPS)

Figure S5 **AKT is involved in vIL-6-induced tumorigenesis promoted by Tat in nude mice. (A)** H&E staining analysis of histological features in tumor tissues from AKT-DN-transfected 4E3 cells followed by Tat transduction in the nude mice. The representative pictures were shown (original magnification, ×200). Arrowheads indicate hemorrhagic foci. **(B)** Immunohistochemical staining analysis of the expression levels of VEGF, b-FGF, cyclin D1, and SMA in tumor tissues from AKT-DN-transfected 4E3 cells followed by Tat transduction in the nude mice. The representative pictures were shown (original magnification, ×400). **(C)** Quantification of results in (B).
(TIF)
