# Supplementary figures and images for: HIV-1 Tat Promotes Kaposi’s Sarcoma-Associated Herpesvirus (KSHV) vIL-6-Induced Angiogenesis and Tumorigenesis by Regulating PI3K/PTEN/AKT/GSK-3β Signaling Pathway
Source: PLoS One. 2013 Jan 2;8(1):e53145. doi: 10.1371/journal.pone.0053145 (PMC3534639; doi:10.1371/journal.pone.0053145)

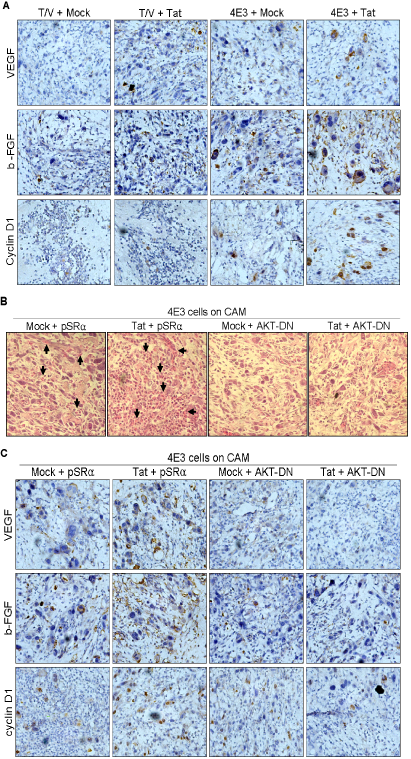

Supplement: Figure S3 — Tat augments vIL-6-induced tumorigenesis, leading to VEGF, b-FGF and Cyclin D1 expression in CAM model. (A) Immunohistochemical staining analysis of the expression levels of VEGF, b-FGF, and cyclin D1 in tumor tissues from Tat-transduced 4E3 cells. The representative pictures were shown (original magnification, ×400). (B) H&E staining analysis of histological features in tumor tissues from AKT-DN-transfected 4E3 cells followed by Tat transduction in the CAM model. The representative pictures were shown (original magnification, ×400). Arrowheads point to hemorrhagic foci. (C) Immunohistochemical staining analysis of the expression levels of VEGF, b-FGF, and cyclin D1 in tumor tissues from AKT-DN-transfected 4E3 cells followed by Tat transduction in the CAM model. The representative pictures were shown (original magnification, ×400). (TIF) [file pone.0053145.s003.tif]

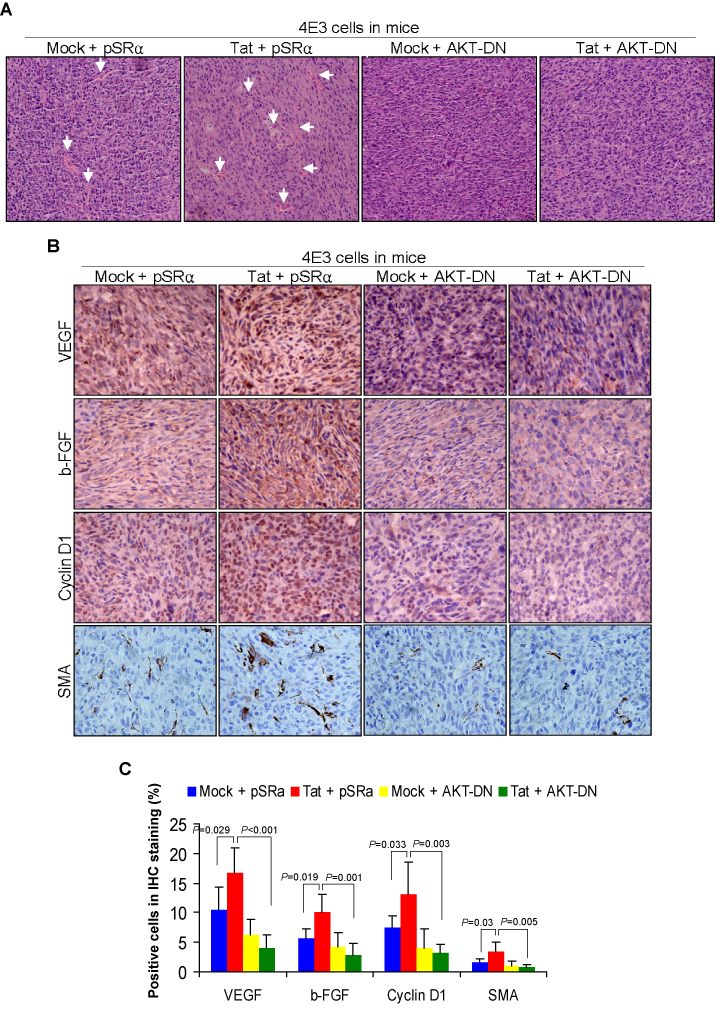

Supplement: Figure S5 — AKT is involved in vIL-6-induced tumorigenesis promoted by Tat in nude mice. (A) H&E staining analysis of histological features in tumor tissues from AKT-DN-transfected 4E3 cells followed by Tat transduction in the nude mice. The representative pictures were shown (original magnification, ×200). Arrowheads indicate hemorrhagic foci. (B) Immunohistochemical staining analysis of the expression levels of VEGF, b-FGF, cyclin D1, and SMA in tumor tissues from AKT-DN-transfected 4E3 cells followed by Tat transduction in the nude mice. The representative pictures were shown (original magnification, ×400). (C) Quantification of results in (B). (TIF) [file pone.0053145.s005.tif]
